# Metabolic Dysfunction Associated Liver Disease in Patients Undergoing Coronary Computed Tomography Angiography

**DOI:** 10.3390/jcdd11030077

**Published:** 2024-02-26

**Authors:** Rares Ilie Orzan, Rares Ioan Gligor, Renata Agoston, Carmen Cionca, Alexandru Zlibut, Raluca Pais, Andrada Seicean, Lucia Agoston-Coldea

**Affiliations:** 1 Department of Internal Medicine, Iuliu Hatieganu University of Medicine and Pharmacy, 400347 Cluj-Napoca, Romania; orzanrares@gmail.com (R.I.O.); luciacoldea@yahoo.com (L.A.-C.); 2 Regional Institute of Gastroenterology and Hepatology “Prof. Dr. Octavian Fodor”, 400162 Cluj-Napoca, Romania; 3 Faculty of Medicine, Iuliu Hatieganu University of Medicine and Pharmacy, 400347 Cluj-Napoca, Romania; 4 Department of Radiology, Affidea Hiperdia Diagnostic Imaging Centre, 400487 Cluj-Napoca, Romania; 5 Institut de Cardiométabolisme et Nutrition, Hôpital Pitié Salpetrière, Assistance Publique Hôpitaux de Paris, 75013 Paris, France; 6 Department of Internal Medicine, Emergency County Hospital, 400347 Cluj-Napoca, Romania

**Keywords:** fatty liver disease, metabolic dysfunction, coronary artery disease, cardiac computed tomography, epicardial fat volume

## Abstract

In this single-center cross-sectional study on patients undergoing coronary computed tomography angiography (CCTA), we assessed the prognostic significance of metabolic dysfunction associated steatotic liver disease (MASLD), metabolic syndrome (MetS), and CCTA-derived parameters for predicting major adverse cardiovascular events (MACE). Over a mean follow-up of 26.9 months, 2038 patients were analyzed, with 361 (17.7%) experiencing MACE. MASLD was associated with a higher MACE incidence (25.90% vs. 14.71% without MASLD, *p* < 0.001). Cox regression revealed significant associations between MASLD, coronary calcium score (CCS), number of plaques (NoP), epicardial fat volume (EFV), and MACE, with hazard ratios of 1.843, 1.001, 1.097, and 1.035, respectively (*p* < 0.001 for all). A composite risk score integrating CCS, NoP, EFV, and MASLD demonstrated superior predictive value for MACE (AUC = 0.948) compared to individual variables (*p* < 0.0001 for all). In conclusion, MASLD is linked to an elevated risk of MACE, and a comprehensive risk-scoring system incorporating imaging and clinical factors enhances MACE prediction accuracy.

## 1. Introduction

Metabolic dysfunction associated steatotic liver disease (MASLD) and metabolic syndrome (MetS) are strongly correlated. In fact, MASLD is often recognized as the hepatic manifestation of MetS due to the close association between the two conditions [1]. Over the past decade, there has been a notable increase in the global prevalence of MASLD, from 25.5% in 2005 to 32.4% in 2021, and it is predicted that it will further increase in the following years, following a similar trend to that of MetS. Consequently, this trend imposes a substantial burden on healthcare services [2].

As MASLD progresses to liver cirrhosis, MASLD is an independent risk factor for major adverse cardiovascular events (MACE), irrespective of other cardiovascular risk factors or the extent of coronary artery disease (CAD) [3,4]. A recent investigation demonstrated that the liver spleen density ratio, employed as an assessment tool for MASLD in patients undergoing coronary computed tomography angiography (CCTA), has emerged as an independent predictor of CAD [5]. Notably, epicardial adipose tissue has been shown to augment coronary atherogenesis, possibly via a paracrine mechanism [6]. Studies have revealed a connection between epicardial fat volume (EFV) and MetS, establishing a correlation with the severity of MASLD. Moreover, it serves as an independent marker for CAD severity, correlating with coronary calcium score (CCS) and aiding in the identification of patients at risk of CCS progression [6,7].

MetS and its components are well-established risk factors for cardiovascular disease. The incidence of MACEs among people diagnosed with MetS is more than four times higher than that among individuals without MetS [8]. Among MetS components, the development of high blood pressure is associated with the highest increase in MACEs incidence, while abdominal obesity and impaired glucose tolerance were associated with a smaller, but still significant risk increase [8]. In Europe, MetS had a prevalence of 24.3%, with slight but significant differences between men and women, and increases with age, reaching a prevalence of more than 30% in those aged 70 or older [9].

Lin et al. showed that native computed tomography (CT)-derived CCS, EFV, and liver attenuation served as predictors for MACE in patients with an intermediate risk of CAD, both with and without MetS [10]. Although the predictive value of CCS is widely acknowledged, it has limitations in estimating MACE risk because of its inability to conclusively identify obstructive coronary artery disease, non-obstructive plaque, or adverse plaque phenotypes, such as low-attenuation plaques. Furthermore, the presence of calcium deposits within the coronary artery signifies an irreversible process linked to plaque stabilization and healing, a phenomenon notably observed in patients undergoing statin therapy [11]. Consequently, CCTA has emerged as the preferred imaging modality for excluding disease in patients with a low to intermediate risk of obstructive CAD.

In this study, our objective was to explore the interplay between MASLD and CCTA-derived features of coronary arteries, specifically CCS and number of coronary plaques (NoP), as well as assessments related to MASLD and EFV. We aimed to discern the connection between these factors and the long-term risk of MACE in patients with suspected CAD.

## 2. Materials and Methods

### 2.1. Study Population

This prospective, single-center, cross-sectional study was conducted at the 2nd Department of Internal Medicine of the Cluj County Emergency Hospital, and data were collected between March 2019 and May 2022. The inclusion criteria comprised consecutive patients suspected of CAD presenting with either typical or atypical chest pain and/or dyspnea, abnormal stress test results, and multiple cardiovascular risk factors [12]. We excluded patients with a history of CAD, prior myocardial infarction, recent acute coronary syndrome, previous percutaneous coronary intervention, coronary artery bypass graft or cerebrovascular disease. Additionally, patients with renal failure, contrast allergy, heavy alcohol consumption, oral corticosteroid or amiodarone use, and a life expectancy of less than one year were also excluded. The study protocol adhered to the principles of the Declaration of Helsinki and was approved by the Ethics Committee of Iuliu Hatieganu University of Medicine and Pharmacy, Cluj-Napoca (decision number 435/15.03.2019).

### 2.2. Medical History and Clinical Examination

We conducted a comprehensive review of various aspects related to the patients, including their demographic information, medical history, cardiovascular risk factors, physical examination findings, electrocardiogram (ECG) results, pre-test probability based on either Duke or updated Diamond–Forrester scores, laboratory test results, stress tests, echocardiograms, and cardiac computed tomography (CT). A change in resting ECG was defined as more than 1-mm ST depression in at least two adjacent leads [12]. Arterial hypertension was defined as systolic blood pressure ≥ 140 mmHg and/or diastolic blood pressure ≥ 90 mmHg or undergoing anti-hypertensive treatment [13]. Diabetes mellitus (DM) was defined as either a fasting plasma glucose level > 126 mg/dL or the use of anti-diabetic therapy [14]. Dyslipidemia was defined as a previous diagnosis of high levels of LDL-C ≥ 140 mg/dL, fasting triglycerides ≥ 150 mg/dL, or the use of lipid-lowering medications [15]. Body mass index (BMI) (calculated as kg/m^2^) was derived from height and weight measurements. Cardiovascular risk was assessed using the ASCVD risk score [16]. Renal function was evaluated based on the estimated glomerular filtration rate (eGFR), and a value below 60 mL/min/1.73 m^2^ indicated impaired renal function [17]. MetS was defined in accordance with a cluster of physiological and biochemical abnormalities, as outlined by the International Diabetes Foundation (IDF) criteria [18]. Specifically, for individuals with a BMI of 30 kg/m^2^ or higher, central obesity was assumed and waist circumference measurement was not required.

### 2.3. Cardiac Computed Tomography

Cardiac CT was performed using a second-generation single-source CT scanner (Siemens SOMATOM Definition Edge, Siemens Healthcare, Erlangen, Germany). The imaging parameters included a 0.6-mm slice thickness and a 1.5-mm reconstruction interval. The tube voltage ranged from 70 to 140 kV with a tube current of 500–650 mAs per rotation. The heart rate adaptive pitch of 0.2–0.5 was employed, and the field of view was adjusted for each patient size. To facilitate contrast injection, a dual-head power injector (SCT 210; Medrad, Warrendale, PA, USA) was used. Non-ionic contrast medium (Omnipaque 350 mgI/mL, GE Healthcare, Princeton, NJ, USA) was administered at a volume of 80–100 mL, followed by saline at a volume of 50–80 mL, delivered at a flow rate of 5 mL/s. Data acquisition commenced 5 s after the threshold of signal attenuation, adhering to current international guidelines [19]. For patients with a heart rate exceeding 65 bpm, prospective ECG triggering was implemented to scan 70 to 80% of the RR-interval. Obstructive CAD was defined as coronary stenosis of >50% [20].

**Figure 1:**
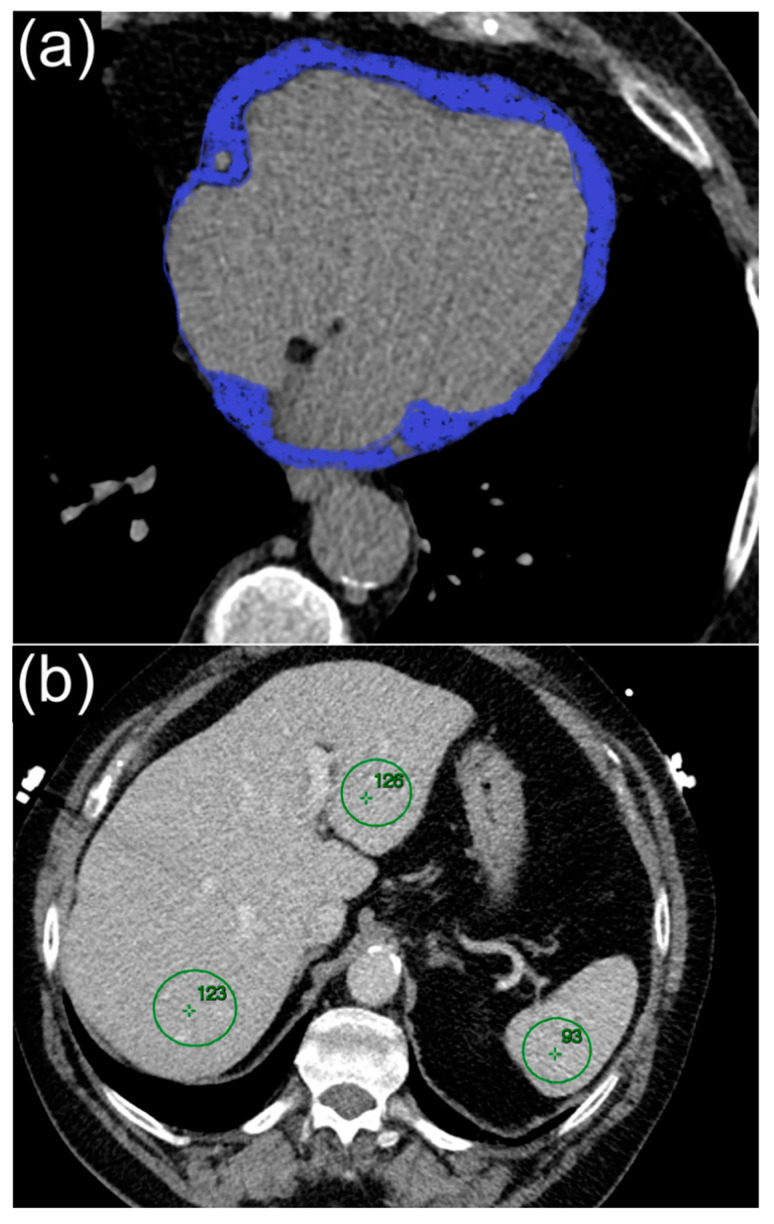
(**a**) Epicardial fat segmentation using semi-automated software. (**b**) Liver and spleen density assessment.

Non-contrast-enhanced scans were obtained at 3 mm slices using a prospective ECG-triggered technique to measure the CCS and EFV. CCS quantification was semi-automatically performed using Syngo Calcium Scoring software (CT VC28, Siemens Healthineers, Erlangen, Germany). The Agatston algorithm was employed for CCS quantification, which was considered significant when a minimum of four contiguous pixels with a density ≥130 Hounsfield Units (HU) and a surface area exceeding 1 mm^2^ were identified [21]. For EFV measurement, slices from the pericardial fat situated 15 mm above and 30 mm below the left main coronary artery were used. This region was chosen to encompass the pericardial fat surrounding the proximity of the coronary arteries. Manual tracing of the pericardial contour was conducted using a cursor pointer on reconstructed axial slices of 0.75 mm thickness. Extrapolation of non-traced slices was performed using specialized software (Syngo Volume, Siemens Medical Solutions). EFV analysis software was then deployed to distinguish fat from other tissues, employing a threshold of −30 to −190 HU (Figure 1) [22]. All examinations were performed by two level III-trained experts, each with over 10 years of experience in advanced cardiovascular imaging and blinded to all clinical data. Discrepancies between the two examiners were resolved by consensus.

### 2.4. Liver Fat Measurement

To evaluate MASLD using CT, the attenuation values in HU of the liver and spleen were quantified by positioning regions of interest (ROIs) greater than 100 mm^2^ within the respective areas. In the same axial section, two ROIs were placed in the right antero-posterior lobe of the liver, one ROI in the left lobe of the liver, and another ROI in the spleen [23]. Subsequently, the HU values obtained from both ROIs of the right lobe of the liver were divided by the HU measurements from the spleen for each subject. This division allowed for calculation of the liver-to-spleen ratio. The mean liver attenuation was determined by averaging the HU values of the ROIs in both the right and left liver lobes. Liver steatosis was defined by a liver-to-spleen ratio of 1.0 and/or a mean liver attenuation of 40 HU or less, while MASLD was defined by its association with one or more cardio-metabolic criteria according to the new Delphi Consensus [24].

### 2.5. Clinical Outcomes

Throughout the follow-up period, patient surveillance was established via various means, including clinical visits, telephone house-calls, and comprehensive questionnaires distributed via mail. The composite endpoint for this study was defined as MACE, which included myocardial infarction (MI), late revascularization, or cardiac death. Hospitalizations unrelated to cardiac causes were not considered in the analytical process.

### 2.6. Statistical Analysis

The data are presented using descriptive statistics, such as mean with standard deviation (SD), median with interquartile range (IQR), or percentage. The chi-square test was used to compare variables across groups. Non-normally distributed variables were log-transformed before the analysis. Based on previous recommendations, CCS was divided into four groups:0, 1–99 (mild), 100–399 (moderate), 400–999 (extensive), and ≥1000 (very extensive). Similarly, for NoPs, patients were classified into four groups: without plaques, 1–5, 6–10 and ≥10. We utilized both unadjusted and multivariable-adjusted models to assess the links between variables and the incidence of MACE: Model 1 (age, sex, MetS), Model 2 (Model 1 + CCS), Model 3 (Model 1 + NoP), Model 4 (Model 1 + EFV), and Model 5 (Model 1 + CCS, NoP, EFV, MASLD). To predict obstructive CAD, we compared the predictive performance of the CCS, EFV, MASLD, and NoP models using Receiver Operating Characteristic (ROC) curves and calculated the Area Under the Curve (AUC). We evaluated the statistical significance and reliability of key determination indices, such as sensitivity, specificity, positive predictive value (PPV), and negative predictive value (NPV). Additionally, we developed a risk-scoring system by utilizing the B coefficients of significant predictors obtained from a logistic regression model, and its effectiveness was assessed using ROC curves. To evaluate event predictions, we employed the Cox regression model and reported outcomes as hazard ratios (HR). Following the analysis of all significant variables in the univariate analysis, we employed a stepwise approach to select the most suitable multivariable models for each outcome. Statistical significance was determined, and event-free survival was determined using the Kaplan-Meier method and log-rank test. The results were considered statistically significant if the *p*-value was less than 0.05. Statistical analysis was conducted using MedCalc, version 19.2.1.

## 3. Results

### 3.1. Baseline Characteristics and CT Measurements

**Figure 2:**
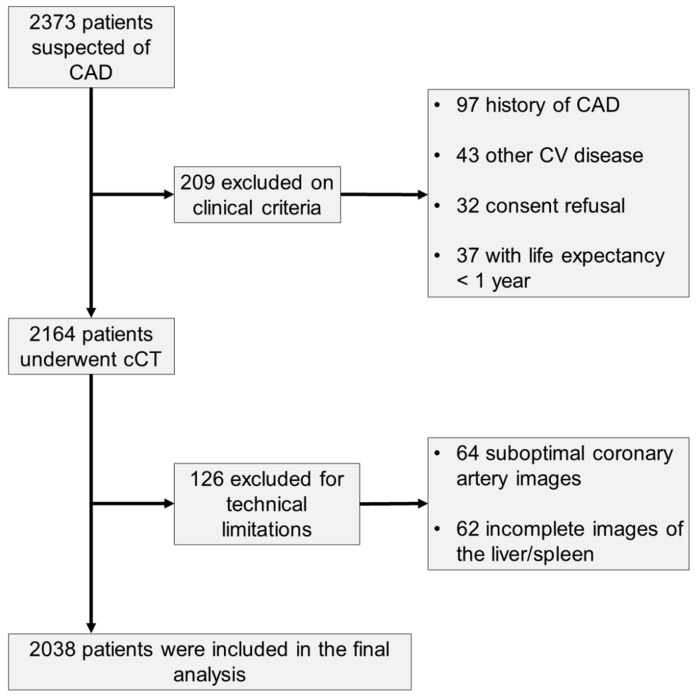
Study design flow chart. CAD was defined as angiographically proven stenosis (>50%) of an epicardial coronary artery; Abbreviations: CAD, coronary artery disease; cCT, cardiac computed tomography; CV, cardiovascular.

The inclusion and exclusion criteria are presented in Figure 2. 2038 patients were followed up over a mean period of 26.9 months (SD: ±9.5). Study participants were categorized into two groups based on the presence or absence of MASLD. The mean age of the enrolled participants was 57.1 (±10.7) years, and no statistically significant differences were observed between these groups. The majority of participants were male, constituting 1060 individuals (52%). Among the patients, 1078 (52.9%) experienced typical angina pectoris, 279 (13.7%) had atypical angina pectoris, 705 (34.6%) presented with non-specific thoracic pain, and 698 (34.2%) reported dyspnea.

Table 1 displays the demographic and clinical characteristics of patients with and without MASLD, along with the levels of serum biomarkers, medication usage, and CCTA-derived parameters.

MetS-positive patients exhibited a notably elevated BMI (*p* < 0.01) and a higher prevalence of dyslipidemia (*p* < 0.001), obesity (*p* < 0.001), arterial hypertension (*p* < 0.001), and smoking (*p* < 0.001) than patients without MetS. Additionally, patients with MetS had significantly higher levels of triglycerides (*p* < 0.001) and blood glucose (*p* < 0.01) and lower levels of HDL-Cholesterol. Furthermore, MetS-positive patients showed higher CCS (*p* < 0.001) and elevated NoP (*p* < 0.001) than MetS-negative patients. The differences between patients with and without MetS are presented in the Supplementary Material (Table S1).

### 3.2. Kaplan–Meier Survival Analysis

On CCTA, 341 patients had coronary stenosis exceeding 50%, of whom 249 had MetS. Following CCTA evaluation, 56 patients required early revascularization. During this monitoring period, 361 patients (17.7%) experienced MACE (myocardial infarction, *n* = 67; late revascularization, *n* = 282; sudden cardiac death, *n* = 12). A higher incidence of MACE was observed among MetS-positive patients (*n* = 244) than among MetS-negative patients (*n* = 117) (*p* < 0.001). Similarly, patients diagnosed with MASLD had a higher MACE incidence rate (25.90%) than those without MASLD (14.71%); (*p* < 0.001).

**Figure 3:**
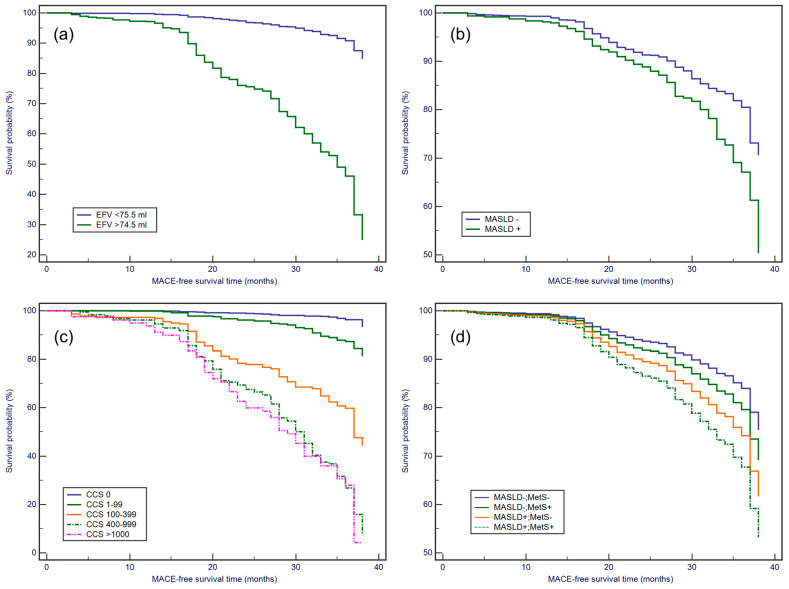
Kaplan–Meier curves of MACE-free survival based on different parameters. (**a**) Epicardial fat volume (EFV). (**b**) Metabolic dysfunction associated steatotic liver disease (MASLD) status. (**c**) Coronary calcium score (CCS) group. (**d**) Metabolic syndrome (MetS) and MASLD status. (Logrank Test, *p* < 0.0001 for all).

Patients with an EFV greater than 74 mL had a higher likelihood of MACE development (Hazard Ratio (HR) = 1.85, 95% CI: 1.50–2.28, *p* < 0.0001), as depicted in Figure 3a. Similar trends were observed for patients with MASLD (HR: 1.67, 95% CI: 1.34–2.07, *p* < 0.0001) (Figure 3b). CCS also emerged as a MACE risk factor, with individuals possessing a CCS greater than zero being at an elevated risk compared with those with a CCS of zero (*p* < 0.0001) (Figure 3c). Individuals with both MetS and MASLD were at a higher risk of developing MACE than those with either MetS or MASLD alone (*p* < 0.001) (Figure 3d).

### 3.3. Association of CCS, NoP, EFV, MASLD and MetS with MACE Risk in Multivariable Cox Regression

Table 2 provides a comprehensive overview of the univariate and multivariate Cox regression analyses, focusing on variables linked to the incidence of MACE. In univariate analysis, factors such as age, male sex, and MetS were significantly associated with MACE occurrence. However, upon incorporating adjustments for CCS, NoP, EFV, and MASLD, the relationship between MetS and the risk of MACE was not statistically significant. Remarkably, all CCTA-derived variables demonstrated robust connections with MACE, substantiated by both univariate and multivariate analyses.

### 3.4. ROC Curve Analysis for Assessing the Ability to Predict MACE

**Figure 4:**
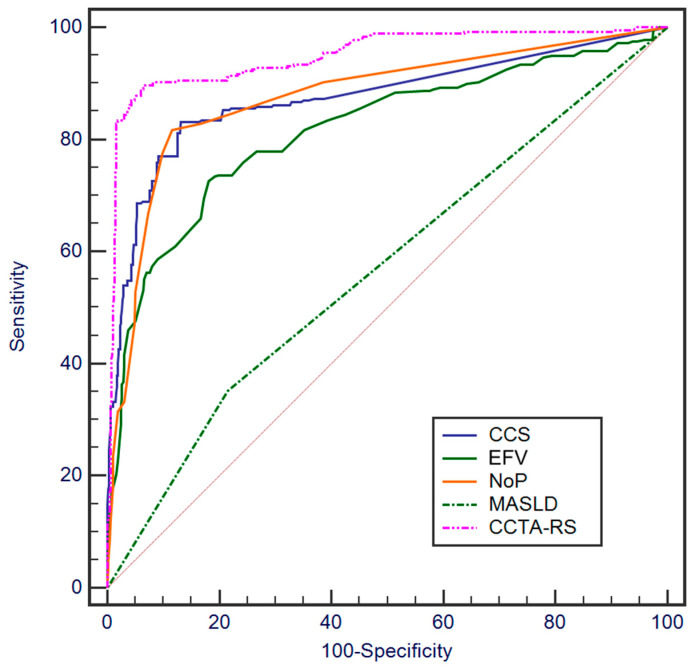
ROC Curves of the included parameters and of the coronary computed tomography angiography derived risk score in predicting MACE (AUC, *p* < 0.001 for all). Abbreviations: CCS, coronary calcium score; EFV, epicardial fat volume; NoP, number of plaques; MASLD, metabolic dysfunction associated steatotic liver disease; CCTA–RS, coronary CT angiography derived risk score.

ROC curve analysis showed that NoP, CCS, EFV, and liver attenuation demonstrated notable predictive capability for the occurrence of MACE, as depicted in Figure 4. The AUC for NoP, CCS, EFV, and MASLD were 0.877, 0.874, 0.817, and 0.568, respectively. For NoP, a cut-off value of 5 yielded a sensitivity of 81.7% and a specificity of 88.4%. In the case of EFV, a sensitivity of 74.5% and specificity of 81.9% were observed for a cut-off value of 74 mL. CCS demonstrated a sensitivity of 83.1% and a specificity of 86.9% at a cutoff value of 70.45 HU. Regarding MASLD status, a sensitivity of 35.2% and specificity of 78.9% were observed.

To identify the most effective predictors for MACEs, we evaluated the AUC of the ROI of the continuous variables. We then selected the variables that exhibited the highest AUC values. Based on this assessment, CCS, NoP, and EFV were selected as key variables for our risk-scoring system. Moreover, a comprehensive approach was adopted wherein the chosen continuous variables, along with categorical variables, such as MASLD, were integrated into a multivariate regression model. Incorporating insights from Cox regression and ROC curve analyses of the selected parameters, we developed a subsequent CCTA-derived risk score for predicting MACE (Equation (S1)).

The ROC curve analysis of our developed risk score for predicting MACE is illustrated in Figure 4. The AUC was 0.948 (95% Confidence Interval (CI): 0.933–0.964) at a defined cut-off value of 11.72. Our CCTA-derived risk score exhibited a sensitivity of 89.75% (95% CI: 86.15–92.68%), a specificity of 92.90% (95% CI: 91.56–94.08%), and a negative predictive value of 97.68% (95% CI: 96.88–98.28%). The overall accuracy of the risk score was 92.34% (95% CI: 91.10–93.46%). CCTA–RS performed better in predicting MACE than each individual variable included (*p* < 0.001 for all), as shown by the ROC curve comparison in Table 3.

The survival analysis of patients categorized as high-risk for MACE using the CCTA-derived risk score revealed a significant variation in survival rates across the groups (log-rank test, *p* < 0.001). Notably, a score of 11.72 or higher was associated with a HR of 35.29 (95% CI: 25.10–49.60).

## 4. Discussion

In our study, which followed symptomatic patients with suspected CAD who underwent CCTA, our primary findings were as follows: 1. MetS was initially associated with a high risk of MACE. However, this association lost statistical significance after adjusting for CCS, NoP, EFV, and MASLD; 2. In addition to CCS, other factors such as EFV, NoP, and MASLD exhibited substantial predictive potential for MACE; 3. The integration of these variables into a novel risk score led to improved classification accuracy for identifying patients at risk of MACE. These results support the EASL recommendations to screen for cardiovascular disease in patients with MASLD, but also suggest that patients at risk for CV disease seen in cardiology should be screened for MASLD and that adding MASLD to classical CV risk factors will improve the prediction of future MACE and eventually improve survival.

The presence of liver steatosis and MASLD has been previously linked to an augmented atherosclerotic burden and elevated risk of MACE. A comprehensive meta-analysis conducted by Targher et al. encompassing 34,043 individuals from 16 studies revealed a remarkable 64% increased risk of fatal and non-fatal cardiovascular events among subjects with MASLD [25]. Remarkably, our study echoes these findings, demonstrating an approximately 67% elevated risk of MACE in individuals with MASLD. The study by Meyersohn et al. conducted in 2021 provided further support for these results [3]. Their investigation illustrated a connection between liver steatosis and the extent of plaque on coronary CCTA, quantified through anatomical measures [3]. Importantly, liver steatosis emerged as an incremental predictor of MACE even after accounting for the burden of coronary artery disease, as well as the presence of high-risk plaques. These findings closely align with those of our study, reinforcing the substantial impact of liver steatosis on cardiovascular risk assessment.

Previous research has demonstrated the utility of CCS in predicting MACE, showing moderate to high sensitivity and specificity across various populations. For instance, the meta-analysis of Kramer et al. showed that CCS has an excellent sensitivity (94%), but a modest specificity (43%) when a cut-off value of 100 HU was used in patients with type 2 DM [26]. Similarly, Kim et al. in 2015 noted a CCS sensitivity of 86% and specificity of 66% for predicting MACE in Korean patients with MetS [27]. Moreover, recent studies have shown a direct link between MetS components, such as elevated blood pressure and triglyceride levels, with CCS progression [28]. In our study, patients with MetS exhibited higher CCS values compared to those without MetS, corroborating these findings.

The NoP observed through CCTA emerged as a valuable indicator of MACE, with patients possessing five or more plaques being associated with an elevated risk for MACE. This aligns with the findings of other studies in this field. For instance, a study conducted by Hoffmann et al. in 2010 reported that patients with three or more plaques detected on CCTA exhibited a significantly heightened risk of MACE when compared to those with two or fewer plaques [29].

Previous research has established that EFV is associated with the occurrence of MACE in asymptomatic patients undergoing native cardiac CT for CCS quantification. The addition of EFV to CCS has shown potential to enhance MACE prediction within this patient group. However, the utility of the EFV assessment in patients undergoing CCTA remains unclear. In our study, we demonstrated that the incorporation of EFV into the predictive framework improves the accuracy of MACE prediction compared with relying solely on CCS in symptomatic patients. The inclusion of EFV alongside CCS, NoP, and MASLD status in a novel risk score has led to a more accurate reclassification of patients at risk of MACE. Interestingly, while previous studies in asymptomatic patients have identified EFV thresholds ranging from 113 to 126.8 mL for MACE prediction [30], our study indicates a notably lower threshold of 74 mL. The distinct symptomatic nature of our patient cohort may account for this discrepancy. While it might be inferred that symptomatic patients are inherently at a heightened risk of future MACE, establishing a direct relationship is challenging due to potential confounding factors such as MetS status, age, and overall cardiovascular risk in the general population. It is pertinent to note that the patients enrolled in our study originated from a region characterized by substantial cardiovascular mortality risk, as classified by the World Health Organization [31]. This context further underscores the relevance and applicability of our findings in the broader clinical landscape.

Our study has several significant strengths, which contribute to its robustness. These strengths encompass a substantial participant cohort, a 26-month mean follow-up period, a notable incidence of MACE during the follow-up period, and a comprehensive evaluation encompassing both MetS status and a range of features derived from CCTA. Nonetheless, it is essential to acknowledge the limitations of this study. First, the absence of a separate validation group for our risk score raises the possibility of overfitting and potential discrepancies in true accuracy. Consequently, the performance of our risk score may not accurately reflect the real-world precision. This study, however, is intended to serve as a foundation for generating hypotheses, necessitating further exploration in future research. Second, the variability in biological marker determination across different laboratories prior to CCTA could serve as a potential confounder influencing the study outcomes. Third, the medication used by each participant was recorded at the moment of the enrolment in the study but not at follow-up, while the revascularization technique (percutaneous coronary revascularization and coronary artery bypass grafting) was not included in our study, making it hard to address the impact of both medical and interventional or surgical treatment on the outcome. Furthermore, it is imperative to acknowledge that our study primarily included symptomatic patients, predominantly of Caucasian ethnicity, from Romania. This specific population context may limit the generalizability of our findings to other demographic groups. While our study has substantial strengths, these limitations warrant consideration when interpreting and extrapolating our results. They underscore the importance of iterative investigations to refine and validate our risk-scoring methodology, along with the necessity to encompass diverse populations for a more comprehensive understanding of its applicability and efficacy.

## 5. Conclusions

MASLD and MetS are associated with a higher risk of MACE. However, MetS association loses statistical significance when accounting for adjustments in CCS, NoP, EFV, and MASLD. CCS, EFV, NoP, and MASLD also exhibit robust predictive potential for MACE. Their inclusion in a novel risk score substantially improved the categorization of patients at risk of experiencing MACE. This underscores the enhanced discriminatory capacity and clinical relevance of our risk score, providing a comprehensive and refined approach to patient risk stratification.

## Figures and Tables

**Table 1:** Baseline characteristics of patients in study grouped by MASLD status.

	All Patients *n* = 2038	MASLD − *n* = 1540	MASLD + *n* = 498	*p*-Value
Demographic characteristics				
- Age, years	57.2 (10.7)	57.0 (10.8)	58.0 (10.5)	0.553
- Male gender, *n* (%)	1060 (52.0)	813 (52.8)	247 (49.6)	0.215
- Body-mass index, kg/m^2^	28.7 (5.5)	27.5 (5.4)	32.4 (5.8)	<0.001
- Systolic blood pressure, mmHg	137.4 (20.1)	136.8 (20.2)	139.2 (19.8)	0.02
- Diastolic blood pressure, mmHg	82.0 (12.1)	81.8 (12.2)	82.5 (11.7)	0.261
- ASCVD Risk Estimator	7.1 (2.7–15.0)	6.9 (2.6–14.8)	8.2 (3.2–15.6)	0.03
CAD risk factors, *n* (%)				
- Hypertension, *n* (%)	1295 (63.6)	980 (63.7)	315 (63.3)	0.877
- Diabetes mellitus, *n* (%)	298 (14.6)	201 (13.0)	97 (19.4)	<0.001
- Dyslipidemia, *n* (%)	1127 (55.3)	743 (48.2)	384 (77.1)	<0.001
- Smoking, *n* (%)	779 (38.2)	565 (36.7)	214 (43.0)	0.01
- Obesity, *n* (%)	1009 (38.1)	667 (43.3)	342 (68.7)	<0.001
- MetS	925 (45.4)	614 (39.9)	311 (62.4)	<0.001
Biomarker levels, mean (SD)				
- Fasting plasma glucose, mg/dL	108.7 (32.4)	107.6 (30.1)	112.1 (38.5)	<0.01
- LDL-Cholesterol, mg/dL	123.4 (42.5)	123.4 (42.9)	123.6 (43.1)	0.928
- HDL-Cholesterol, mg/dL	45.9 (14.7)	46.4 (14.3)	44.3 (15.9)	<0.01
- Triglyceridemia, mg/dL	166.1 (28.9)	165.1 (28.1)	169.1 (31.2)	<0.01
- eGFR, mL/min/1.73 m^2^	90.2 (23.2)	90.8 (23.3)	88.7 (22.6)	0.078
Medications, *n* (%)				
- Beta-blockers, *n* (%)	1515 (74.4)	1134 (73.7)	381 (76.5)	0.202
- ACEIs or ARBs, *n* (%)	978 (48.0)	722 (46.9)	256 (51.4)	0.079
- Calcium channel blockers, *n* (%)	401 (19.7)	321 (20.9)	80 (16.1)	0.02
- Statins, *n* (%)	928 (45.5)	590 (38.3)	338 (67.8)	<0.001
- Antiplatelet therapy, *n* (%)	425 (20.8)	301 (19.5)	124 (24.9)	0.01
- Diuretics, *n* (%)	520 (25.5)	390 (25.3)	130 (26.2)	0.728
Coronary Computer Tomographic Angiography				
- CCS, HU	4.6 (0.0–109.7)	2.9 (0.0–102.1)	16.1 (1.4–118.6)	0.01
- CCS Group				
0	805 (39.5)	681 (44.2)	124 (24.9)	<0.001
1–99	543 (26.6)	408 (26.5)	135 (27.1)	0.787
100–399	324 (15.9)	206 (13.4)	118 (23.7)	<0.001
400–999	286 (14.1)	194 (12.6)	92 (18.5)	0.001
>1000	80 (3.9)	51 (3.3)	29 (5.8)	0.01
- NoP				
0	776 (38.1)	611 (39.7)	165 (33.2)	0.01
1–5	514 (25.2)	366 (23.8)	148 (29.7)	<0.01
6–10	692 (34.0)	545 (35.4)	147 (29.5)	0.02
>10	56 (2.7)	18 (1.1)	38 (7.6)	<0.001
- EFV, mL	64.1 (20.4)	63.5 (20.2)	65.9 (21.1)	0.02
- Liver attenuation, UH	63.3 (7.7)	65.0 (6.9)	59.6 (8.5)	<0.01

Abbreviations: *n*, number of patients; IQR, interquartile range; ASCVD, atherosclerotic cardiovascular disease; eGFR, estimated glomerular filtration rate; ACEI, angiotensin converting enzyme inhibitor; ARB, angiotensin receptor blocker; CAD, coronary artery disease; CCS, coronary calcium score; EFV, epicardial fat volume; HDL-C, high-density lipoprotein cholesterol; LDL-C, low-density lipoprotein cholesterol; MASLD, metabolic dysfunction associated steatotic liver disease; MetS, metabolic syndrome; NoP, number of calcified plaques. Values are expressed as *n* (%), mean +/− standard deviation or median (interquartile range, 25th–75th).

**Table 2:** Multivariable Cox regression for Predictor of MACEs.

	Univariate	Multivariate				
		Model 1	Model 2	Model 3	Model 4	Model 5
Age	1.25 (1.15–1.35) (*p* < 0.001)	1.03 (1.02–1.04) (*p* < 0.001)	1.011 (1.001–1.021) (*p* = 0.03)	1.003 (0.993–1.013) (*p* = 0.51)	1.01 (1.00–1.03) (*p* = 0.02)	1.005 (0.995–1.014) (*p* = 0.36)
Male gender	1.87 (1.51–.2.31) (*p* < 0.001)	2.23 (1.32–3.77) (*p* = 0.003)	1.57 (0.93–2.67) (*p* = 0.09)	1.43 (0.84–2.42) (*p* = 0.18)	1.56 (0.92–2.63) (*p* = 0.09)	1.297 (0.761–2.211) (*p* = 0.34)
MetS	1.56 (1.13–1.98) (*p* < 0.001)	1.16 (0.68–1.98) (*p* = 0.59)	1.21 (0.71–2.07) (*p* = 0.49)	1.19 (0.69–2.03) (*p* = 0.52)	1.08 (0.63–1.85) (*p* = 0.78)	1.05 (0.61–1.79) (*p* = 0.86)
CCS	1.0013 (1.0012–1.0014) (*p* < 0.001)	–	1.0012 (1.0011–1.0013) (*p* < 0.001)	–	–	1.001 (1.000–1.001) (*p* < 0.001)
NoP	1.16 (1.15–1.18) (*p* < 0.001)	–	–	1.16 (1.14–1.18) (*p* < 0.001)	–	1.097 (1.075–1.119) (*p* < 0.001)
EFV	1.053 (1.047–1.058) (*p* < 0.001)	–	–	–	1.05 (1.04–1.06) (*p* < 0.001)	1.035 (1.030–1.041) (*p* < 0.001)
MASLD	1.67 (1.34–2.07) (*p* < 0.001)	–	–	–	–	1.843 (1.475–2.303) (*p* < 0.001)
*p*-value Hosmer–Lemeshow		<0.001	<0.0001	<0.001	<0.001	<0.0001

Abbreviations: CAD, coronary artery disease; CCS, coronary calcium score; EFV, epicardial fat volume; NoP, number of plaques; HU, Hounsfield units; MASLD, metabolic dysfunction associated steatotic liver disease. Data are hazard ratios (95% CI). Model 1 = age + sex + MetS; Model 2 = Model 1 + CCS; Model 3 = Model 1 + NoP; Model 4 = Model 1 + EFV; Model 5 = Model 1 + CCS + NoP + EFV + MASLD.

**Table 3:** ROC Curves comparison between CCTA–RS and the individual parameters.

CCTA–RS vs.	Δ AUC	*p*
NoP	0.072 (0.058–0.086)	<0.001
CCS	0.075 (0.058–0.091)	<0.0001
EFV	0.132 (0.106–0.158)	<0.0001
MASLD	0.381 (0.347–0.415)	<0.0001

Abbreviations: CCS, coronary calcium score; EFV, epicardial fat volume; NoP, number of plaques; MASLD, metabolic dysfunction associated steatotic liver disease.

## Data Availability

Not available.

## Supplementary Materials

The following supporting information can be downloaded at https://www.mdpi.com/article/10.3390/jcdd11030077/s1. Table S1: Baseline characteristics of patients in study grouped by metabolic syndrome (MetS) status; Equation (S1): CCTA-RS.

## Abbreviations

ASCVD	Atherosclerotic Cardiovascular Disease
AUC	area under the curve
BMI	body mass index
CAD	coronary artery disease
CCS	coronary calcium score
CCTA	coronary computed tomography angiography
CT	cardiac computed tomography
DM	diabetes mellitus
ECG	electrocardiogram
EFV	epicardial fat volume
eGFR	estimated glomerular filtration rate
HDL-C	high-density lipoprotein cholesterol
HU	Hounsfield Units
IQR	interquartile range
LDL-C	low-density lipoprotein cholesterol
MACEs	major adverse cardiovascular events
MASLD	metabolic dysfunction associated steatotic liver disease
NoPs	number of coronary plaques
NPV	negative predictive value
PPV	positive predictive value
ROC	receiver operating characteristics
Se	sensitivity
Sp	specificity
