# Supplementary material for: Metabolic Dysfunction Associated Liver Disease in Patients Undergoing Coronary Computed Tomography Angiography
Source: J Cardiovasc Dev Dis. 2024 Feb 26;11(3):77. doi: 10.3390/jcdd11030077 (PMC10971240; doi:10.3390/jcdd11030077)
Supplement: Supplementary file 1 [file jcdd-11-00077-s001.zip › jcdd-2865779-supplementary.pdf]

Metabolic dysfunction associated steatotic liver disease in patients undergoing coronary computed tomography angiography

Rares Ilie Orzan[1,2,*], Rares Ioan Gligor[1], Renata Agoston[3], Carmen Cionca[1,4], Alexandru Zlibut[1], Raluca Pais[5], Andrada Seicean[1,2], Lucia Agoston-Coldea[1,4,6]

[1]Department of Internal Medicine, Iuliu Hațieganu University of Medicine and Pharmacy, 400347 Cluj-Napoca, Romania.
[2]Regional Institute of Gastroenterology and Hepatology, Prof. Dr. Octavian Fodor, 400162 Cluj-Napoca, Romania.
[3]Faculty of Medicine, Iuliu Hatieganu University of Medicine and Pharmacy, 400347 Cluj-Napoca, Romania.
[4]Department of Radiology, Affidea Hiperdia Diagnostic Imaging Centre, 400487 Cluj-Napoca, Romania.
[5]Institut de Cardiométabolisme et Nutrition, Hôpital Pitié Salpetrière, Assistance Publique Hôpitaux de Paris, 75013 Paris, France.
[6]Department of Internal Medicine, Emergency County Hospital, 400347 Cluj-Napoca, Romania.

***) Corresponding author**

Iuliu Hatieganu University of Medicine and Pharmacy
E-mail: orzanrares@gmail.com

Table of Contents

Table S1 2
Equation S1 3

1. Tables

Table S1. Baseline characteristics of patients in study grouped by metabolic syndrome (MetS) status

		All patients n=2038	MetS - n=1113	MetS+ n=925	*p-Value*
Demographics characteristics					
- Age, years		57.2 (10.7)	56.7 (11.1)	57.8 (10.1)	NS
- Male gender, n (%)		1060 (52.0)	578 (51.9)	482 (52.1)	NS
- Body-mass index, kg/m^2		28.7 (5.5)	23.6 (3.5)	34.8 (4.2)	<0.001
- Systolic blood pressure, mmHg		137.4 (20.1)	132.1 (20.4)	143.8 (22.1)	<0.001
- Diastolic blood pressure, mmHg		82.0 (12.1)	79.3 (11.9)	85.2 (12.5)	<0.001
- ASCVD (%)		7.1 (2.7-15.0)	4.2 (1.9-8.4)	11.3 (4.9-21.5)	<0.001
CAD risk factors, n (%)					
- Hypertension, n (%)		1295 (63.9)	667 (59.9)	628 (67.9)	<0.001
- Diabetes mellitus, n (%)		298 (14.6)	112 (10.1)	186 (20.1)	<0.001
- Dyslipidemia, n (%)		1127 (55.3)	466 (41.9)	661 (71.5)	<0.001
- Smoking, n (%)		779 (38.2)	275 (24.7)	504 (54.5)	<0.001
- Obesity, n (%)		1009 (49.5)	84 (7.5)	925 (100)	<0.001
Biomarker levels, mean (SD)					
- Fasting plasma glucose, mg/dl		108.7 (32.4)	104.2 (28.4)	114.0 (36.7)	<0.001
- LDL-Cholesterol, mg/dl		123.4 (42.5)	122.6 (43.2)	124.4 (41.5)	NS
- HDL-Cholesterol, mg/dl		45.9 (14.7)	47.1 (14.7)	44.5 (14.6)	<0.001
- Triglyceridemia, mg/dl		166.1 (28.9)	148.9 (28.8)	186.7 (29.3)	<0.001
- eGFR, ml/min/1.73 m^2		90.2 (23.2)	92.3 (23.2)	87.7 (22.9)	NS
Medications, n (%)					
- Beta-blockers, n (%)		1515 (74.4)	821 (73.8)	694 (75.0)	NS
- ACEIs or ARBs, n (%)		978 (48.0)	413 (37.1)	565 (61.0)	<0.001
- Calcium channel blockers, n (%)		401 (19.7)	221 (19.8)	180 (19.4)	NS
- Statins, n (%)		928 (45.5)	422 (37.9)	506 (54.7)	<0.001
- Antiplatelet therapy, n (%)		425 (20.8)	176 (15.8)	249 (26.9)	<0.01
- Diuretics, n (%)		520 (25.5)	289 (25.9)	231 (24.9)	NS
Coronary Computer Tomographic Angiography					
- CCS, median (25th–75th)		4.6 (0-109.7)	3.3 (0.0-105.1)	10.5 (0.0-136.7)	<0.001
- Group CCS	0	805 (39.5)	504 (45.3)	301 (32.6)	<0.001
	1-99	543 (26.6)	282 (25.3)	261 (28.2)	NS
	100-399	324 (15.9)	159 (14.3)	165 (17.8)	0.03
	400-999	286 (14.1)	154 (13.8)	132 (14.3)	NS
	>1000	80 (3.9)	14 (1.3)	66 (7.1)	<0.001
- NoP					
	0	776 (38.1)	422 (37.9)	354 (38.3)	NS
	1-5	514 (25.2)	269 (24.2)	245 (26.5)	NS
	6-10	692 (34.0)	400 (35.9)	292 (31.6)	0.05
	>10	56 (2.7)	22 (2.0)	34 (3.6)	0.05
- EFV, ml		64.1 (20.4)	62.3 (19.5)	66.3 (20.9)	<0.001
- Liver attenuation, UH		63.3 (7.7)	65.4 (7.9)	60.7 (7.3)	<0.001
- MASLD		498 (24.4)	187 (16.8)	311 (33.6)	<0.001

Abbreviations: n, number of patients; IQR, *interquartile range*; eGFR, estimated glomerular filtration rate; ACEI, angiotensin converting enzyme inhibitor; ARB, angiotensin receptor blocker; ASA, acetylsalicylic acid; CAD, coronary artery disease; CCS, coronary calcium score; EFV, epicardial fat volume; HDL-C, high-density lipoprotein cholesterol; LDL-C, low-density lipoprotein cholesterol; LVEDV, left ventricular end-diastolic volume; LVESV, left ventricular end-systolic volume; LVM, left ventricular mass; LVEF, left ventricular ejection fraction; MASLD, metabolic dysfunnctionn associated steatotic liver disease; MetS, metabolic syndrome; NoP, number of calcified plaques.

Values are expressed as n (%), mean +/- standard deviation or median (*interquartile range, 25th-75th)*

2. **Equation S1**. CCTA-RS formula.

$$\mathbf{CCTA - RS = \ln MASLD * (CCS + EFV) * (NoP + 1)}$$

Where MASLD =1.684 if MASLD+, or 1 if MASLD-

CCTA-RS, CCTA-derived risk score; NoP, number of plaques; CCS, coronary calcium score (HU); EFV, epicardial fat volume
